# Photothermal-enhanced catalysis in core–shell plasmonic hierarchical Cu_7_S_4_ microsphere@zeolitic imidazole framework-8†
†Electronic supplementary information (ESI) available: Experimental details, calculation of photothermal transduction efficiency, additional analytical data, spectra, and images. See DOI: 10.1039/c6sc03239g



**DOI:** 10.1039/c6sc03239g

**Published:** 2016-08-11

**Authors:** Feifan Wang, Yanjie Huang, Zhigang Chai, Min Zeng, Qi Li, Yuan Wang, Dongsheng Xu

**Affiliations:** a Beijing National Laboratory for Molecular Sciences , State Key Laboratory for Structural Chemistry of Unstable and Stable Species , College of Chemistry and Molecular Engineering , Peking University , Beijing 100871 , P. R. China . Email: dsxu@pku.edu.cn; b Academy for Advanced Interdisciplinary Studies , Peking University , Beijing 100871 , P. R. China

## Abstract

Conventional semiconductor photocatalysis based on band-edge absorption remains inefficient due to the limited harvesting of solar irradiation and the complicated surface/interface chemistry. Herein, novel photothermal-enhanced catalysis was achieved in a core–shell hierarchical Cu_7_S_4_ nano-heater@ZIF-8 heterostructures *via* near-infrared localized surface plasmon resonance. Our results demonstrated that both the high surface temperature of the photothermal Cu_7_S_4_ core and the close-adjacency of catalytic ZIF-8 shell contributed to the extremely enhanced catalytic activity. Under laser irradiation (1450 nm, 500 mW), the cyclocondensation reaction rate increased 4.5–5.4 fold compared to that of the process at room temperature, in which the 1.6–1.8 fold enhancement was due to the localized heating effect. The simulated sunlight experiments showed a photothermal activation efficiency (PTAE) of 0.07%, further indicating the validity of photothermal catalysis based on the plasmonic semiconductor nanomaterials. More generally, this approach provides a platform to improve reaction activity with efficient utilization of solar energy, which can be readily extended to other green-chemistry processes.

## Abstract 2

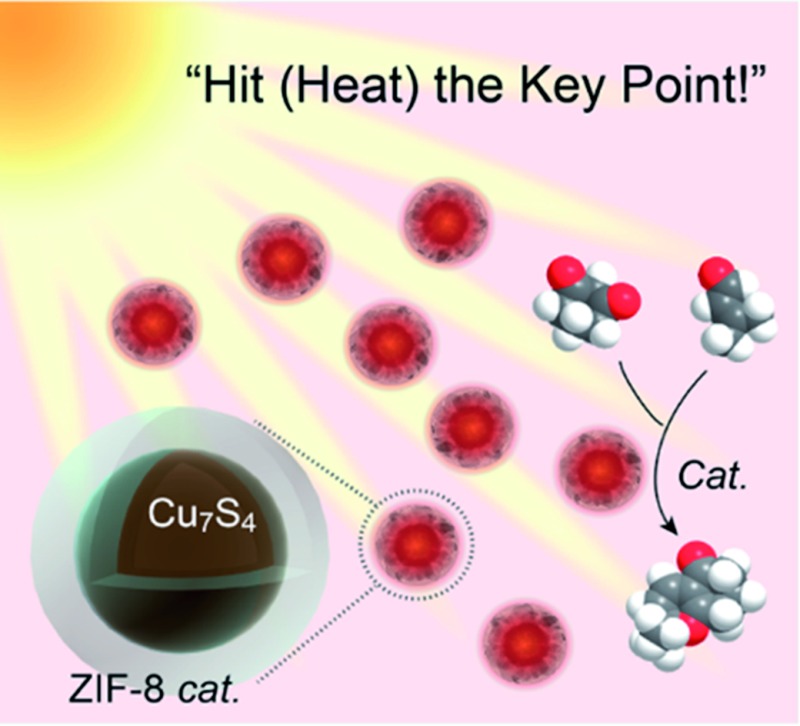
A strategy to improve reaction activity *via* the photothermal effect of plasmonic semiconductor nanomaterials is demonstrated in a core–shell structured catalyst.

## Introduction

Semiconductor nanomaterials have been widely investigated as photocatalysts for the direct conversion of solar energy to chemical energy in the fields of water photolysis, CO_2_ conversion, and organic photosynthesis.^1–4^ The general pathway is to utilize photo-induced electrons and holes to initiate or accelerate specific redox reactions through the bandgap absorption of the semiconductor. However, this strategy remains inefficient due to two major challenges: (i) narrow bandgap semiconductors tend to exhibit efficient optical absorption, but their band-edge positions are usually incompatible with the electrochemical potentials to trigger specific redox reactions; (ii) surface/interface modifications are expected to improve the catalytic activity of the nanocatalysts and thus, unfortunately, introduce surface defects into the system, which would increase the recombination of the photo-excited electrons and holes. For instance, difficulties in energy band configuration limit sunlight utilization to mainly within the ultraviolet and visible range, resulting in near-infrared light (NIR, ∼54% of solar radiation at the Earth's surface) unable to be harvested.^5^

An alternative approach for efficient conversion of solar energy is based on the photothermal effect of nanoparticles (NPs) to generate hot carriers to participate in redox reactions, or to dissipate the absorbed photon energy into heat to accelerate the reaction rate.^6–10^ Comparing the two mechanisms, the heating pathway is supposed to be a versatile strategy due to lower requirements of the surface properties of the catalyst. According to collision theory, the proportion of reactant molecules with energy larger than the activation energy (*E* > *E*_a_) increases after absorbing heat, bringing about more successful collisions and a corresponding increase in reaction rate. Furthermore, the high temperature gradient in the vicinity of the surface of photothermal materials can promote the diffusion of reactants and products, especially for microporous catalysts.^11^

The photothermal effect induced by the localized surface plasmon resonance (LSPR) of noble metal NPs has been widely used in photothermal therapy, chemical synthesis, targeted delivery, photothermal evaporation, and photoacoustic imaging.^12–16^ In recent studies, plasmonic semiconductor materials have shown evident advantages over noble metals for harvesting NIR light due to their low cost, sufficient abundance, high extinction coefficient and wide-range tunable LSPR band.^17–21^ Particularly, copper chalcogenide nanocrystals have been demonstrated to exhibit a comparable photothermal performance to gold NPs and have shown good performance for the photothermal ablation of cancer cells.^22^ Moreover, delicate designs of the nanostructures provided the opportunity to further enhance the NIR absorption of these semiconductor materials, such as the collective effect of nanoparticle assemblies and the optical cavity of hollow nanostructures.^23,24^ Consequently, copper chalcogenide nanomaterials with specific structures are promising candidates for developing photothermal catalysts with efficient solar-to-chemical energy conversion.^25^

**Scheme 1:**
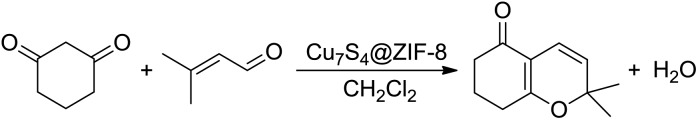
Model photothermal catalysis based on the cyclocondensation reaction.

To integrate dual photothermal- and catalytic-functionality, a core–shell heterostructure should be a plausible model. Although core–shell nanostructures based on NPs and metal organic frameworks (MOFs) have been investigated, to the best of our knowledge, only a few have reported desirable results in the application of catalysis.^26–36^ Just recently, Q. H. Yang, *et al.* have reported that the Pd nanocubes@zeolitic-imidazolate-framework (ZIF-8) composite showed efficient hydrogenation of olefins based on the catalytic and visible-light plasmonic properties of noble-metal Pd nanocubes.^30^ Herein, we develop a versatile strategy to accelerate chemical reactions based on the NIR plasmonic photothermal effect of a low-cost semiconductor nanomaterial with a well-defined core–shell Cu_7_S_4_@ZIF-8 nanostructure. We demonstrated that the core and shell section of the composite was endued with photothermal and catalytic functionality, respectively. The hierarchical Cu_7_S_4_ hollow microsphere core acts as a plasmonic nano-heater with a photothermal transduction efficiency of 31.1% and the surface temperature increased up to 94.0 °C under the irradiation of a 1450 nm laser. The localized heat converted from light energy directly acts on the surrounding ZIF-8 shells with synergistic acid–base catalytic sites (the proper positioned Zn(ii) and imidazolate functional groups), which leads to improved catalytic activity for the valuable cyclocondensation reaction (Scheme 1).^37,38^

## Results and discussion

### Controllable synthesis of core–shell structures and hierarchical nano-heaters

**Fig. 1:**
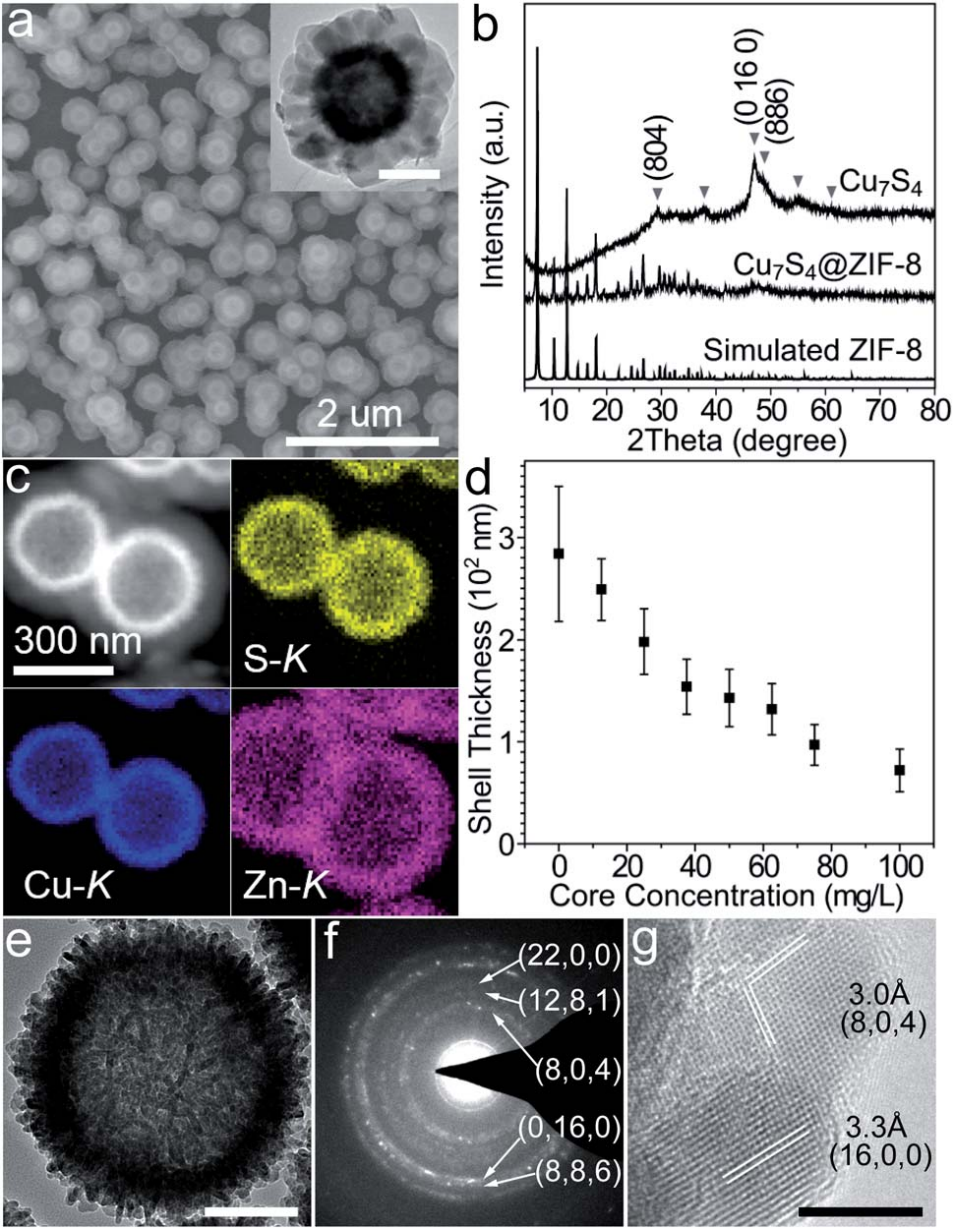
(a) Scanning electron microscopy (SEM) and transmission electron microscopy (TEM) (inset) images of the core–shell Cu_7_S_4_@ZIF-8 NPs. (b) X-ray diffraction (XRD) patterns of simulated ZIF-8, Cu_7_S_4_@ZIF-8 and Cu_7_S_4_ NPs. (c) HAADF-STEM image and EDX elemental mapping of the core–shell nanostructures. (d) Shell thickness as a function of core concentration. (e) TEM image and (f) SAED pattern of individual hierarchical Cu_7_S_4_ hollow microspheres. (g) HRTEM image of nanocrystal building blocks of the Cu_7_S_4_ NP. Scale bar: 200 nm, inset of (a); 100 nm, (e); 5 nm, (g).

Typically, hierarchical Cu_7_S_4_ hollow microspheres were pre-synthesized from the Cu_2_O nanosphere precursors using established methods and their surfaces were modified by PVP during the preparation (Fig. S1†).^39,40^ The encapsulation procedure involves the rapid mixing of methanolic solutions of zinc nitrate (10 mM, 150 mL), 2-methylimidazole (10 mM, 150 mL) and Cu_7_S_4_ NPs (10 g L^–1^, 1.5 mL) at room temperature standing for 2 h without stirring. Fig. 1a shows the representative SEM and TEM images of the core–shell structures. The grayscale contrast of the core and shell indicates the successful encapsulation of Cu_7_S_4_ NPs by ZIF-8 with the thickness of ∼120 nm. THe powder XRD pattern (Fig. 1b) displays two sets of peaks, which are assigned to the monoclinic phase of Cu_7_S_4_ (JCPDS 23-958) and cubic phase of ZIF-8. The elemental mapping of Cu, S, and Zn (Fig. 1c) further testifies the formation of a core–shell structure. Notably, almost no unencapsulated particles are observed (Fig. 1a and S2†), indicating dominant heterogeneous nucleation around the nanoparticles. This is attributed to the appropriate crystallization conditions, including the proper solvent polarity, surface properties of NPs, and the dosage of MOF precursors and NPs.^27,28^ Both the coordination interaction between pyrrolidone groups (C=O) and zinc ions and the hydrophobic interaction between apolar chains of PVP and organic linkers lead to the fact that the ZIF-8 shell exclusively grows on the Cu_7_S_4_ microsphere cores.^27^ Additionally, the concentration of NPs is also important to form homogeneous core–shell structures with the desired shell thickness. Fig. 1d shows that the shell thickness can be controlled from 72 ± 21 to 249 ± 30 nm with the overall core concentration decreasing from 100 to 12.5 mg L^–1^ (see also Fig. S3 in ESI†). Insufficient core concentrations result in the homogeneous nucleation of ZIF-8 and excessive ones cause aggregation of the core–shell NPs. All of the above indicates that the encapsulation of ZIF-8 around Cu_7_S_4_ NPs is both a thermodynamically- and kinetically-controlled process.

Fig. 1e shows that the hierarchical Cu_7_S_4_ hollow microsphere is composed of nanoplate subunits with diameter of 19.8 ± 3.5 nm and thickness of 6.2 ± 1.2 nm, formed by the sulfuration of polycrystalline Cu_2_O nanoparticles. The lattice spacing of 3.0 Å and 3.3 Å (Fig. 1g) can be indexed to the (8, 0, 4) and (16, 0, 0) planes of the roxbyite Cu_7_S_4_ structure, respectively. The selected area electron diffraction (SAED) pattern (Fig. 1f) reveals that the diffraction rings consisted of scattered dots, in accordance with the nature of crystalline nanoplates and polycrystalline microspheres. These Cu_7_S_4_ nanocrystal subunits have been confirmed to support the LSPR mode due to abundant carrier concentration caused by copper deficiencies.^20^ Thus, the hierarchical structure is expected to show efficient light absorption over a large spectral range.

### Light-harvesting property and bulk photothermal effect

**Fig. 2:**
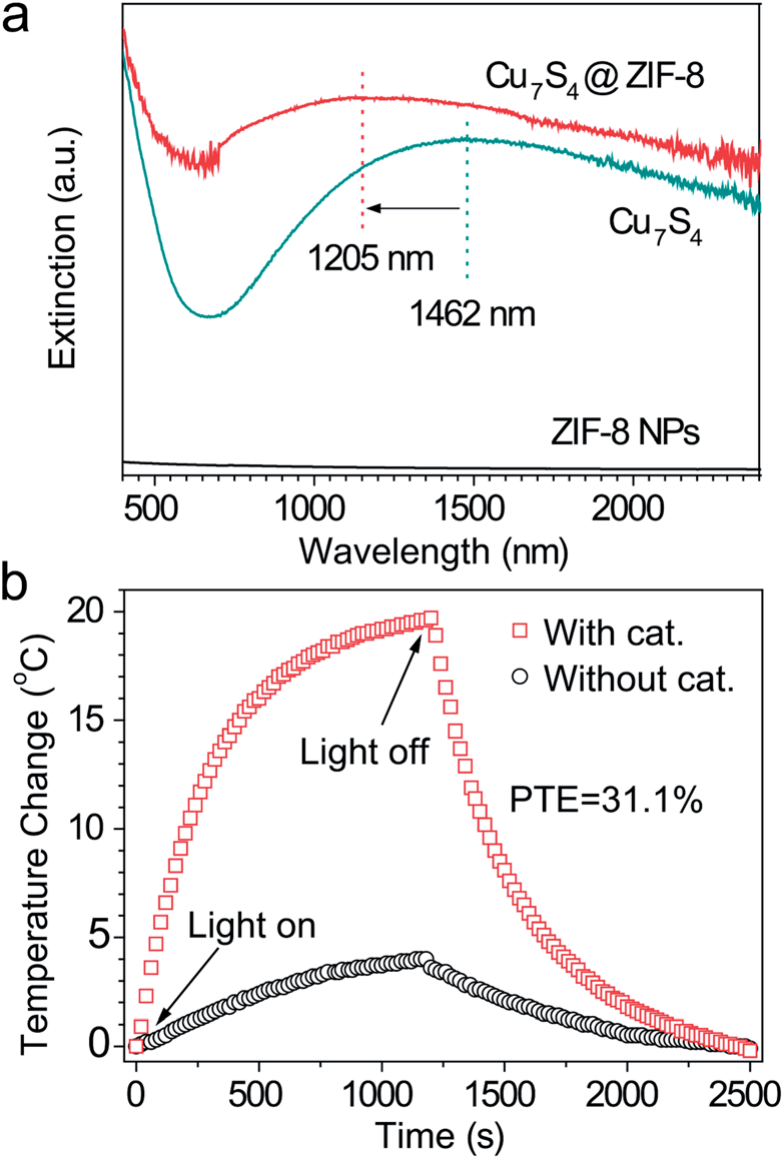
(a) Extinction spectra of Cu_7_S_4_, ZIF-8, and Cu_7_S_4_@ZIF-8 NPs. The spectra were collected by dispersing the samples in a KBr matrix. (b) Steady state heating data (bulk temperature) of the dichloromethane solution with or without the Cu_7_S_4_@ZIF-8 NPs irradiated by a 1450 nm laser light with an intensity of 500 mW cm^–2^.

Fig. 2a shows a broad extinction peak in the NIR range for the hierarchical Cu_7_S_4_ hollow microspheres, while the copper sulfide nanocrystals usually exhibit a relatively sharp peak within this range.^25,41^ This may result from the collective effect of plasmonic nanocrystals and the optical cavity of the hollow structures. Compared with Cu_7_S_4_ NPs, the core–shell Cu_7_S_4_@ZIF-8 NPs show a blue-shift of the plasmonic band due to the decrease of dielectric constant from 2.43 (KBr) to 1.8 (ZIF-8).^42,43^ Interestingly, the core–shell structure showed enhanced absorption properties as compared with the sum of individual ZIF-8 and Cu_7_S_4_ components. It is reasonable to deduce that there exist optical synergistic effects between Cu_7_S_4_ and ZIF-8, which is of benefit for light-harvesting. The photothermal transduction efficiency *η* of Cu_7_S_4_@ZIF-8 NPs (2.5 g L^–1^) was measured by illuminating the nanostructure dispersion to a steady-state temperature increase (Δ*T*).^44,45^ The value of *η* = 31.1% and Δ*T*_max_ = 19.8 °C are extracted from the thermal profile (Fig. 2b) *via* the energy balance function
1

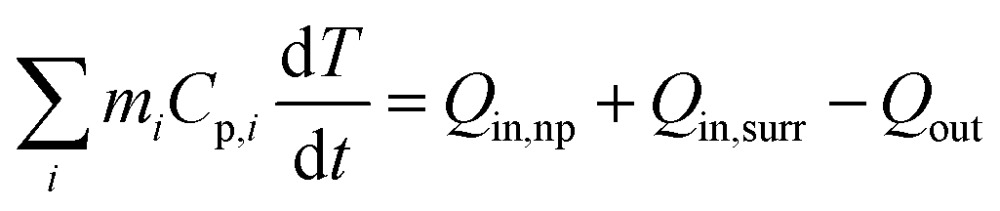

where *m*_*i*_ and *C*_p,*i*_ are the mass and heat capacity of system components, *Q*_in,np_, *Q*_in,surr_, and *Q*_out_ are the heat input from the nanostructures, surroundings and the heat lost term, respectively. A detailed derivation is shown in the ESI.† The temperature profile without containing the Cu_7_S_4_@ZIF-8 NPs (Fig. 2b) shows a much smaller temperature increase and a slower dynamic response, indicating the heating effect is mainly attributed to the photothermal properties of the Cu_7_S_4_@ZIF-8 nanostructures.

### Measurement of surface temperature of the nano-heaters

**Fig. 3:**
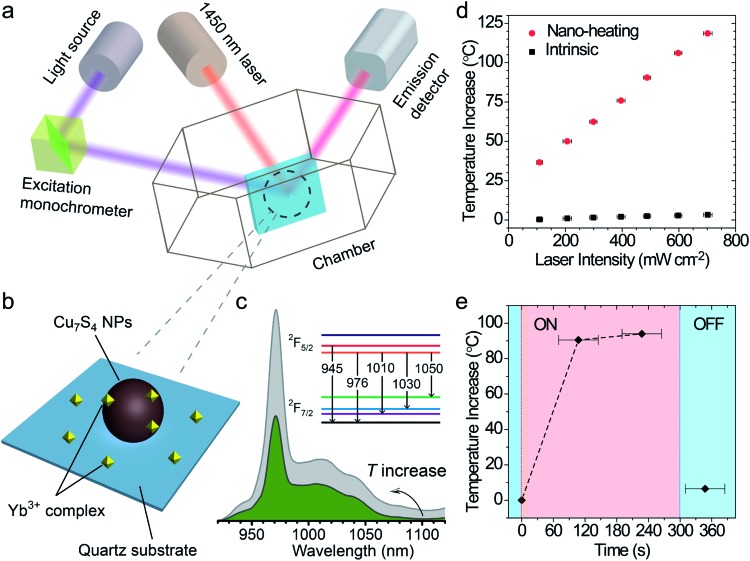
(a) Schematic depiction of a surface temperature measurement of Cu_7_S_4_ nanostructures based on temperature-sensitive fluorescent molecules. The 410 nm laser was used to excite the fluorescent molecule and a 1450 nm laser was used as the heating source. (b) Magnified diagram of the testing sample on the quartz substrate. The ytterbium(iii) complex was absorbed on the surface of Cu_7_S_4_ NPs as a temperature sensor. (c) *T*-Dependent luminescence spectra of the Yb^3+^ complex, with the inset showing the energy level corresponding to the luminescent wavelength. (d) Surface temperature increase (Δ*T*) of Cu_7_S_4_ NPs (circle) and the sensor (square) at different intensities of 1450 nm laser illumination for 1 min. (e) On/off response of the surface temperature increase of Cu_7_S_4_ NPs.

The surface temperature increase (also called “local temperature”) of the Cu_7_S_4_ NPs was measured by using an ytterbium(iii) complex as the temperature sensor,^46^ which was absorbed on the surface of Cu_7_S_4_ NPs (schematic in Fig. 3a–c). The integral area ratio between the luminescence spectrum branches of the complex shows a good linear relationship towards the temperature (Fig. S4†). The surface Δ*T* increases from 37.0 °C to 122.0 °C with the laser intensity increasing from 100 mW cm^–2^ to 700 mW cm^–2^ (as shown in Fig. 3d and S5, Table S1†), while the intrinsic Δ*T* of the sensor does not change much under each intensity of laser irradiation. It is reasonable to think such a high local-temperature is favorable for the acceleration of reaction rate. The thermogravimetry–differential thermal analysis (Fig. S6†) shows no obvious weight-loss up to 190 °C under an air atmosphere, which confirms that the thermal stability of the core–shell structures can be guaranteed under the present photothermal conditions. The on/off response of surface Δ*T* of Cu_7_S_4_ NPs was also measured with the irradiation of the 1450 nm laser. Fig. 3e shows that the surface Δ*T* of the nano-heater increased to 90.5 °C in ∼2 min and 94.0 °C in ∼4 min (light on) and decreased to 6.5 °C within several tens of seconds (light off). The rapid *T*-transformation may provide us with a convenient way to switch the reaction by introduction or removal of light flux.

### Laser experiments of photothermal catalysis and calculation of photothermal activation efficiency

**Fig. 4:**
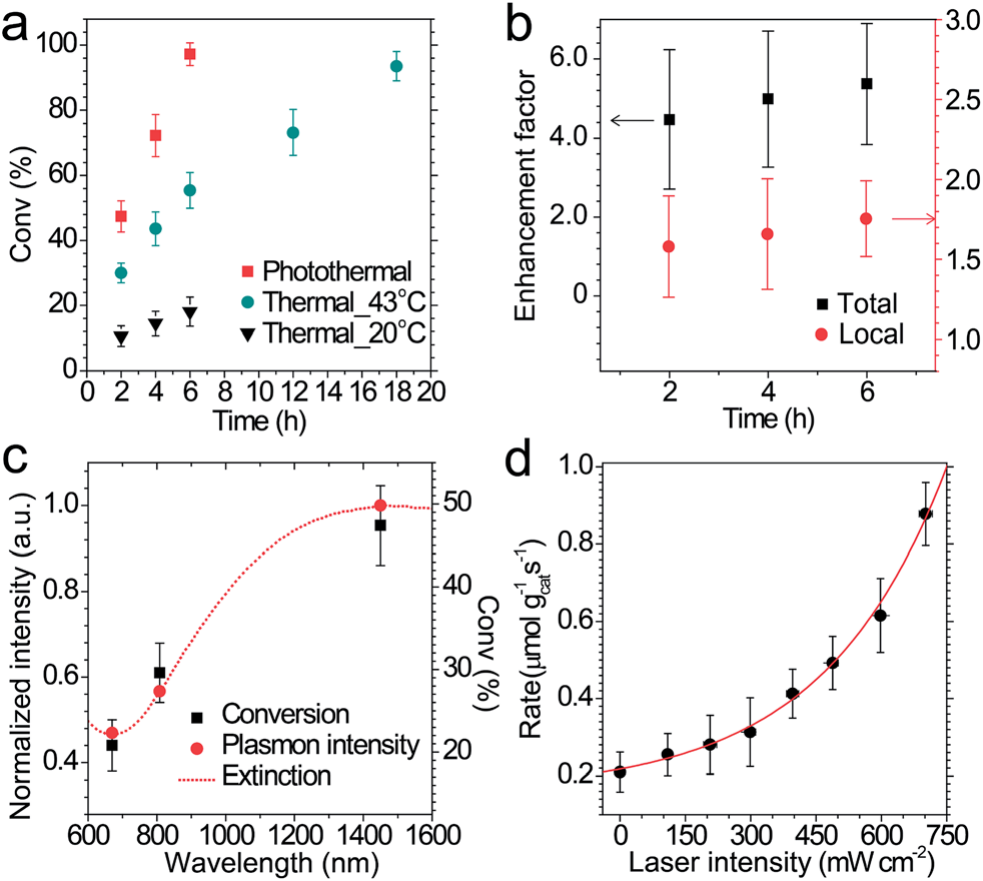
(a) Conversion efficiency of the cyclocondensation reaction *via* photothermal and thermal processes as a function of time. The photothermal catalysis was conducted under laser irradiation (1450 nm, 500 mW cm^–2^) without thermostatic control and the highest temperature of the bulk reaction solution was 43.3 °C. (b) Total and local conversion enhancement factor calculated by dividing the conversion rate of the photothermal route by that of the thermal route at 20 °C and 43 °C, respectively. (c) First 2 hour catalysis performance and normalized plasmon intensity at different laser wavelengths. The dashed line shows the experimental extinction of the NPs. (d) Initial rate of photothermal catalysis at different laser intensities.

The performance of photothermal catalysis of the core–shell Cu_7_S_4_@ZIF-8 NPs was evaluated using the cyclocondensation reaction of 1,3-cyclohexanedione and 3-methyl-2-butenal (Scheme 1). The reaction can occur at the external surface of the crevices of the polycrystalline shell,^37,47–49^ where the substrate can directly sense the temperature of the photothermal core. Furthermore, the small-sized solvent molecule, CH_2_Cl_2_, can freely diffuse in the micropores of ZIF-8 and act as the carrier for heat transfer in the thin shell, which the substrates can indirectly feel the temperature of the core. Reasonably, our results showed an evident enhancement of the catalytic performance through the photothermal effect (Fig. 4) and supported the above assumptions. The highest temperature of the bulk reaction solutions containing Cu_7_S_4_@ZIF-8 NPs was 43.3 °C (Fig. S7†) with the 1450 nm laser irradiation (500 mW). Fig. 4a shows the conversion efficiency of photothermal catalysis reached 97.2% within 6 h, while that of the thermal catalysis under constant *T* of 43 °C and 20 °C was 55.4% and 18.1%, respectively, while the other conditions remained the same (also in Table S2†). An efficiency of 93.5% was obtained with constant *T* = 43 °C for 18 h, indicating the conventional heating method was a time-consuming process. Fig. 4b showed the conversion enhancement factor of photothermal-to-thermal catalysis at different times. The introduction of the photothermal effect showed a 4.5–5.4 fold total enhancement, with a 1.6–1.8 fold local enhancement coming from the high local temperature in the vicinity of the nano-heater core. The possible reason is that the heating of the Cu_7_S_4_ core not only establishes a high *T* environment to increase the activity of reactants, but also supplies a large *T* gradient to facilitate the product diffusion in the crevices of the MOF shell. Fig. 4c shows the catalytic activity of core–shell NPs under 670, 808 and 1450 nm laser irradiation with the same intensity. The plasmon intensity was calculated by multiplying the laser intensity by the measured wavelength-dependent extinction.^50^ The positive correlation between the conversion efficiency and the normalized plasmon intensity revealed that the observed enhancement was related to the excitation of the surface plasmon. Fig. 4d shows an exponential relationship between the reaction rate and the laser intensity, following the Arrhenius empirical equation
2

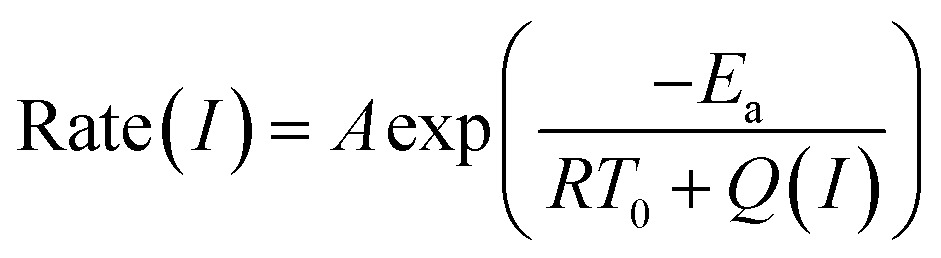

where *I* is the light intensity, *E*_a_ is the apparent activation energy, *A* is the pre-exponential factor, and *Q*(*I*) is the plasmonic heating energy *via* photothermal effect. The mechanism of energetic electrons or holes was unreasonable in this situation due to the dielectric nature of the ZIF-8 shell. Therefore, the plasmon-induced photothermal effect is responsible for the enhanced catalytic activity. Here, we propose a concept of photothermal activation efficiency (PTAE) to describe the utilization efficiency of light energy for the activation of chemical reactions *via* the photothermal effect, which is defined as
3

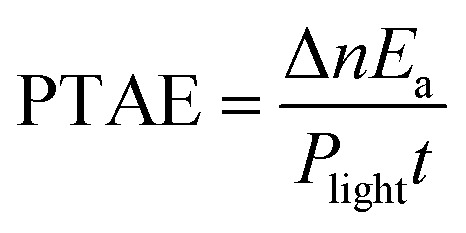

where Δ*n* is the increased amount of chemical reactions caused by the photothermal effect, *P*_light_ is the incident light power, and *t* is the illumination time. The reaction enthalpy (Δ*H*) and *E*_a_ was estimated theoretically and experimentally to be –370 kJ mol^–1^ and 107 kJ mol^–1^, respectively (Fig. S8 and S9 and Table S3†). The PTAE was calculated to be 0.08% for the cyclocondensation reaction under the 1450 nm laser irradiation (substrate-to-catalyst amount ratio of 0.01 mol g^–1^).

### Full-solar-spectrum photothermal mechanism and simulated sunlight catalytic performance

**Fig. 5:**
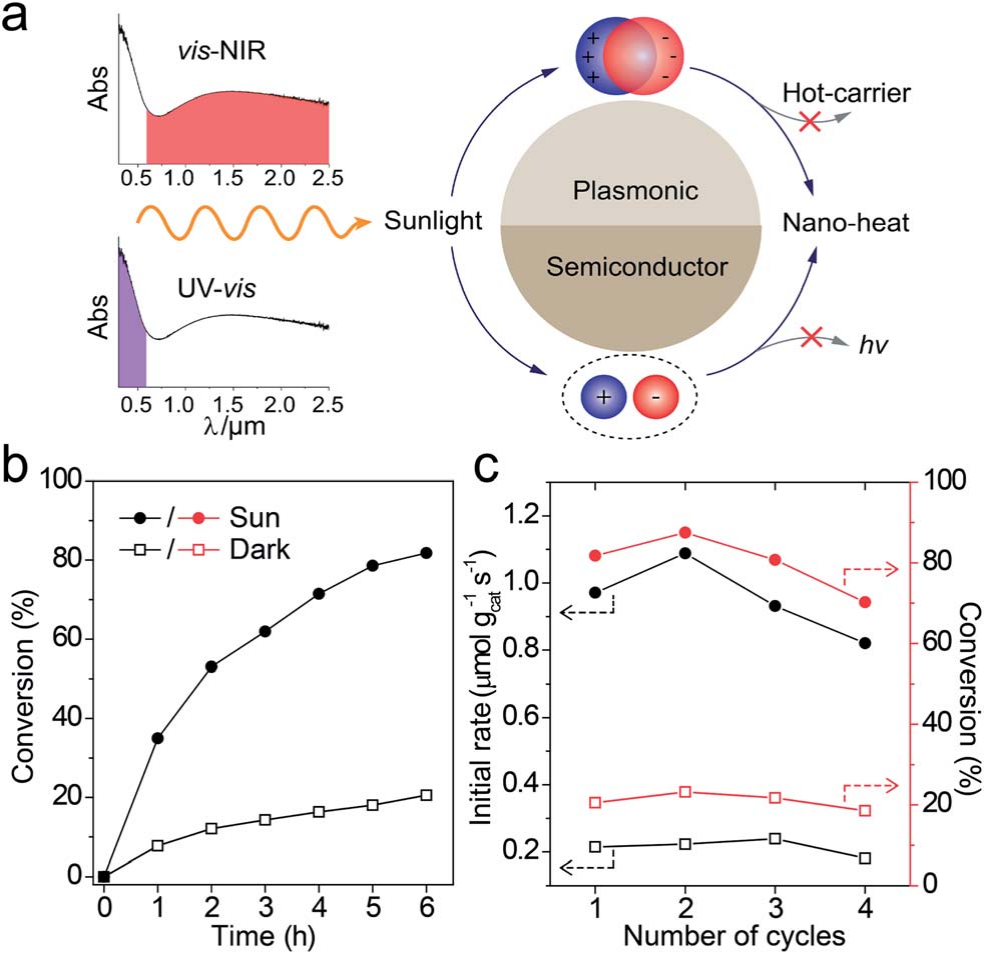
(a) Schematic depiction of the full-solar-spectrum photothermal effect of plasmonic semiconductor nanomaterials *via* plasmon- and exciton-based approaches. (b) Catalytic performance of Cu_7_S_4_@ZIF-8 NPs with or without the simulated sunlight illumination. (c) Recyclability test (1st hour rate and 6 hour conversion) of the core–shell photothermal catalyst. Reaction conditions: 100 mW cm^–2^ full-spectrum irradiation, 11.2 cm^2^ illumination area, 25 mL reactor, room temperature.

The catalytic reactions were further performed under the simulated sunlight irradiation to verify the pragmatic feasibility of our photothermal catalysts. Fig. 5a illustrates that the hierarchical Cu_7_S_4_ nanoparticles can harvest full-spectrum solar energy due to their semiconductor and plasmonic characteristics. Fig. S10† shows the poor luminescence properties of the Cu_7_S_4_ NPs with a quantum yield <0.1%, indicating that the radiative decay pathway is blocked. Additionally, the plasmon decay *via* energizing charge carriers is also inhibited because of the dielectric nature of the ZIF-8 shell. Therefore, the core–shell NPs are supposed to exhibit excellent photothermal effect due to the dominant heating decay pathway of the absorbed solar energy. Fig. 5b shows the kinetic analysis of the cyclocondensation reaction of the core–shell NPs under simulated sunlight or dark conditions. An efficiency of 81.8% was obtained within 6 h in the former case, while 20.5% was obtained for the reaction conducted in the dark. The apparent enhancement was ascribed to the efficient conversion of sunlight energy to heat of the photothermal core, which was directly executed on the adjacent catalytic shell. The PTAE for the cyclocondensation reaction was calculated to be 0.07% under the simulated sunlight illumination with a substrate-to-catalyst amount ratio of 0.01 mol g^–1^ (Fig. S11†). This effective photothermal conversion arises from both the excitation of localized surface plasmon in the vis-NIR range and the band-gap absorption in the UV-vis range. The recyclability of the core–shell catalysts was also tested (Fig. 5c). After four consecutive runs, the Cu_7_S_4_@ZIF-8 with the simulated sunlight irradiation still showed evident enhancement than that in the dark (4.4 fold in initial rate and 3.8 fold in final conversion). This is consistent with the XRD characterizations (Fig. S12†) that two sets of peaks of the core–shell structure can be preserved after the recycling experiments. The gradual decrease in the long-term test of the photothermal catalysis may be caused by the corrosion of ZIF-8 by H_2_O formed during the reaction process (Fig. S13†). The UV-vis-NIR spectra showed that the Cu_7_S_4_@ZIF-8 NPs maintain a high extinction coefficient in the NIR range (Fig. S14†), testifying the good optical stability of these nanostructures. Obviously, our photothermal catalysis pathway based on the core–shell plasmonic-semiconductor@MOF nanostructure is desirable for high-efficiency and time-saving catalytic applications.

## Conclusions

In summary, we have successfully synthesized a core–shell Cu_7_S_4_ NPs@ZIF-8 heterostructure that is rationally designed with a plasmonic photothermal core and a catalytic shell. Through the intense LSPR absorption of the hierarchical Cu_7_S_4_ hollow microsphere, the ZIF-8 shell showed an enhanced conversion efficiency for the cyclocondensation reaction. The laser experiments revealed that the plasmon-induced photothermal effect is responsible for improved catalytic activity and the simulated sunlight experiments further indicated this effect is valid for high-efficiency and time-saving catalysis. Particularly, the rapid *T*-transformation at the surface of the NPs provides the opportunity to switch the chemical reaction on/off with fast response speed. From the perspective of application, this strategy is expected to construct a localized high-temperature environment under industry-favored ambient conditions, facilitating the practical conversion of solar energy to chemical energy.
