# Supplementary material for: Photothermal-enhanced catalysis in core–shell plasmonic hierarchical Cu7S4 microsphere@zeolitic imidazole framework-8
Source: Chem Sci. 2016 Aug 11;7(12):6887–93. doi: 10.1039/c6sc03239g (PMC5450595; doi:10.1039/c6sc03239g)
Supplement: Supplementary file 1 [file SC-007-C6SC03239G-s001.pdf]

Electronic Supplementary Material (ESI) for Chemical Science.
This journal is © The Royal Society of Chemistry 2016

Electronic Supplementary Information

Photothermal-Enhanced Catalysis in Core-Shell Plasmonic Hierarchical Cu_7S_4 Microsphere@Zeolitic Imidazole Framework-8

Feifan Wang,[ab] Yanjie Huang,[a] Zhigang Chai,[a] Min Zeng,[a] Qi Li,[a] Yuan Wang,[a] and Dongsheng Xu*[ab]

[a]Beijing National Laboratory for Molecular Sciences, State Key Laboratory for Structural Chemistry of Unstable and Stable Species, College of Chemistry and Molecular Engineering, Peking University, Beijing 100871, P. R. China

[b]Academy for Advanced Interdisciplinary Studies, Peking University, Beijing 100871, P. R. China

Keywords: copper sulfide, heterogeneous catalysis, metal-organic frameworks, photothermal effect, plasmonic semiconductor

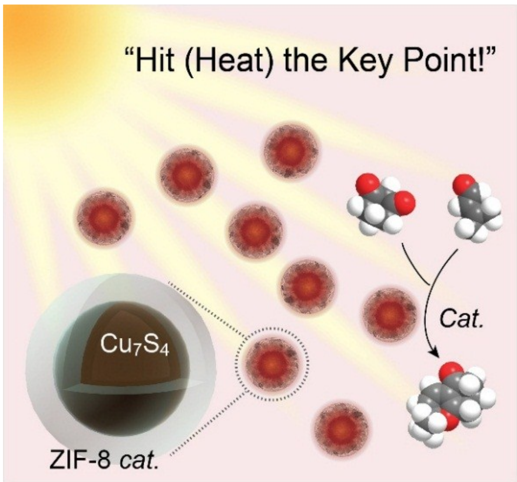

*Correspondence and requests for materials should be addressed to Dongsheng Xu (email: dsxu@pku.edu.cn)

Experimental details

Materials. Copper nitrate hemi-pentahydrate ($Cu(NO_3)_2·2.5H_2O$, 98%) was purchased from Alfa Aesar (USA). Aqueous ammonium sulfide ($(NH_4)_2S$, 20 wt %), zinc nitrate ($Zn(NO_3)_2·6H_2O$, 99%) and 2-methylimidazole (99%) was obtained from J&K Chemicals (China). Polyvinylpyrrolidone (PVP-K30, average MW 40,000), diethylene glycol (DEG, 99%), 1,3-cyclohexanedione (97%) and 3-methyl-2-butenal (97%) were purchased from Aldrich (USA). Dichloromethane (99.9%), methanol (99.9%), ethanol (99.9%) and acetone (99.9%) were supplied by Tianjin Concord Co., Ltd (China).

Preparation of hierarchical Cu_7S_4 hollow microspheres. The hierarchical Cu_7S_4 hollow microspheres were pre-synthesized from the Cu_2O nanosphere precursors using established methods.[1,2] Briefly, 2.5 mmol $Cu(NO_3)_2·2.5H_2O$ and 6 g PVP-K30 were dissolved in 80 mL DEG at 60°C with an oil-bath heating. Then the dark green solution was degassed at 100°C for 15 min and the flask was purged with argon and kept under an argon atmosphere during the whole synthesis. The temperature was increased to 200°C at a rate of 5°C/min with the color changing to pale orange. After maintained at 200°C for 60 min, the mixture was cooled to room-temperature and centrifuged at 10000 rpm for 5 min. Then the Cu_2O NPs were washed three times with ethanol/acetone solvent and re-dispersed in 30 mL ethanol. The sulfuration of Cu_2O NPs was used $(NH_4)_2S$ as the sulfur precursor. A certain amount of aqueous $(NH_4)_2S$ solution was added in the above Cu_2O solution with the Cu:S ratio of 1:1. The reaction was conducted at room-temperature for 45 min to form a dark brown

color. The suspension was centrifuged at 6000 rpm for 5 min and washed three times with ethanol. The Cu_7S_4 NPs was dried in a vacuum oven at 60°C for 12 h and then fully dispersed in methanol to form a solution with concentration of 10 mg/mL.

Encapsulation of hierarchical Cu_7S_4 hollow microspheres with ZIF-8. The methanolic zinc nitrate (10 mM) and 2-methylimidazole (10 mM) were prepared as the precursors of ZIF-8. The Cu_7S_4 NPs suspension (1.5 mL) was first mixed with 150 mL 2-methylimidazole solution. 150 mL zinc nitrate solutions was quickly added into the above mixture and kept at room-temperature for 2 h without stirring. Then the products were centrifuged at 8000 rpm for 5 min and washed with methanol for three times. The as-synthesized core-shell NPs were immersed in CH_2Cl_2 for 12 h to replace the absorbed methanol in the pores of ZIF-8. Finally, the samples were dried at 60°C for 12 h before the catalytic evaluation.

Characterizations. The morphology and composition of the Cu_7S_4 and core-shell Cu_7S_4@ZIF-8 nanostructures were characterized by field-emission scanning electron microscopy (Hitachi, S-4800), transmission electron microscopy (Tecnai F20, FEI). The crystalline structure of the NPs was determined by a powder X-ray diffraction (Rigaku, D/MAX-2500 diffractometer with Cu Kα radiation). Extinction spectra of the Cu_7S_4, ZIF-8 and Cu_7S_4@ZIF-8 NPs were obtained using a UV-vis-NIR spectrometer (Hitachi, U4100). The thermogravimetric analysis (TGA) was carried out on a Q600 SDT instrument (TA, USA) with a ramping rate of 10.0°C/min in 100 mL/min of air flow. The luminescent spectra of the ytterbium complex and the Cu_7S_4 NPs were collected on a lifetime and steady state spectrometer (Edinburgh

Instruments Ltd., FLS920). The temperature of the bulk reaction solution was measured by a K/J type thermometer (Suzhou Tasi Electronic Co., Ltd, TASI-602). The monochromatic light sources were 670, 808, 1450 nm diode laser with a fiber optic accessory (Beijing Hi-Tech Optoelectronic Co., Ltd) and the output power was calibrated by an optical power meter (Ophir Optronics Solutions Ltd, Vega). The sunlight photothermal catalysis was conducted under the simulated solar irradiation with intensity of 100 mW cm^{-2} obtained by a 500 W Xenon lamp with the AM 1.5G filter (Beijing Au-Light Tech Co., Ltd, CEL-S500R).

Catalyst evaluation. The cyclocondensation reaction was conducted in a 10 mL rubber-sealed flask under atmospheric pressure. The reactions (**Figure 4**, **S11**) were performed in a mixture of 1,3-cyclohexanedione (0.1 mmol), 3-methyl-2-butenal (0.1 mmol) and dichloromethane (2 mL) with the amount of catalysts adjusted to contain 10 mg ZIF-8. The measurement of the reaction extent was based on the conversion efficiency of 1,3-cyclohexanedione, whose amount was analyzed by gas chromatographs (Agilent, 7890B). All reactions conducted in our experiments performed a 99.9% selectivity with the Cu_7S_4@ZIF-8 catalyst, which was consistent with the previous report.[3] For the determination of the relationship between photothermal catalytic activity and the laser intensity, the initial reaction rate was calculated based on the conversion efficiency of the first hour. The recyclability test of the Cu_7S_4@ZIF-8 (with or without the sunlight irradiation) were performed in a mixture of 1,3-cyclohexanedione (0.6 mmol), 3-methyl-2-butenal (0.6 mmol) and dichloromethane (12 mL) with the amount of catalysts adjusted to contain 60 mg ZIF-

8. The catalyst was centrifuged at 6000 rpm for 5 min after each cycle of the 6-hour catalysis. The supernatant liquid was decanted slowly and the precipitate was washed with CH_2Cl_2 and immersed in CH_2Cl_2 for 12 h and centrifuged at 6000 rpm for 5min. Then the precipitate was dried in vacuum at 60°C for at least 6 h. Then, the catalyst was reused with the injection of fresh solvent and reactants for the next catalytic cycle under the same reaction conditions.

Surface temperature measurement of Cu_7S_4 nano-heaters. The measurement of surface temperature was based on the temperature-sensitive fluorescent molecules, an Yb^{3+} complex. The 410 nm laser was used to excite the fluorescent molecule and the 1450 nm laser was used as the heating source. To prepare the testing sample, the methanolic solutions of 1 mL Yb^{3+} complex (1 g/L) and 1 mL Cu_7S_4 NPs (2 g/L) were mixed and standed for 5 min to ensure the sufficient absorption of the Yb^{3+} complex on the surface of the Cu_7S_4 NPs. Then the mixture (~100 μL) was dispensed on the quartz substrate using a pipet and dried in air. The luminescence spectrum of the Yb^{3+} *T*-sensor was collected from 900nm to 1150 nm (step length: 1.0 nm, dwell time: 0.3 s, scan number: 1). The value of the surface temperature was determined according to the standard curve.

Measurement of photothermal transduction efficiency (PTE) of core-shell nanostructures. The photothermal transduction efficiency η of Cu_7S_4@ZIF-8 NPs was measured by illuminating the nanostructure dispersion to a steady-state temperature increase.[4,5] **Figure 2**b shows a typical thermal profile of the sample

system. The energy balance of the entire system can be described as

$$\sum_i m_i C_{\mathrm{p},i} \frac{dT}{dt} = Q_{\mathrm{in,np}} + Q_{\mathrm{in,surr}} - Q_{\mathrm{out}} \quad (1)$$

where m_i and $C_{\mathrm{p},i}$ are the mass and heat capacity of system components (solvent and glass sample cell). $Q_{\mathrm{in,np}}$ is the heat input from the nanostructures via photothermal effect

$$Q_{\mathrm{in,np}} = I\left(1-10^{-A_\lambda}\right)\eta \quad (2)$$

where η is the photothermal transduction efficiency, A_λ = 6.3 is the absorbance at the excitation wavelength of 1450 nm (measured by UV-vis-NIR spectrascopy), and I = 500 mW is the laser power. $Q_{\mathrm{in,surr}}$ is the heat input due to the light absorption of the borosilicate glass cell (C_{glass} = 0.840 J g^{-1} K^{-1}, m_{glass} = 2.5 g) containing dichloromethane (C_{CH2Cl2} = 102.3 J mol^{-1} K^{-1}, ρ_{CH2Cl2} = 1.325 g mL^{-1}, V_{CH2Cl2} = 2.0 mL) and is measured independently to be 57.3 mW.

$$Q_{\mathrm{in,surr}} = \frac{\Delta T \cdot \left(C_{\mathrm{glass}} \cdot m_{\mathrm{glass}} + C_{\mathrm{CH_2Cl_2}} \cdot \rho_{\mathrm{CH_2Cl_2}} \cdot V_{\mathrm{CH_2Cl_2}} / M_{\mathrm{CH_2Cl_2}}\right)}{t}$$

$$= \frac{13 \times \left(0.840 \times 2.5 + 102.3 \times 1.325 \times 2.0 / 84.93\right)\mathrm{J}}{60 \times 20\mathrm{s}} = 57.3\ \mathrm{mW}$$

The heat lost term, Q_{out}, is depicted as

$$Q_{\mathrm{out}} = hA\left(T - T_{\mathrm{surr}}\right) \quad (3)$$

where T_{surr} is the surrounding temperature, h is a heat-transfer coefficient, and A is the surface area of the glass container. At the maximum temperature (T_{max}), the system heat flux reaches a steady-state that the laser-induced energy input equals to the energy transfer out of the system:

$$Q_{\mathrm{in,np}} + Q_{\mathrm{in,surr}} = Q_{\mathrm{out}} \quad (4)$$

Substituting eq 2 and 3 into eq 4, we get

$$\eta = \frac{hA\left(T_{\max} - T_{\text{surr}}\right) - Q_{\text{in,surr}}}{I\left(1 - 10^{-A_{\lambda}}\right)} \tag{5}$$

From the results deduced by Roper and co-workers,[4] the temperature increase as a function of time during the laser irradiation can be expressed as

$$\frac{T_{amb} - T}{T_{amb} - T_{\max}} = 1 - \exp\left(-t/\tau_{\text{s}}\right) \tag{6}$$

by introducing a system time constant τ_{s}

$$\tau_{\text{s}} \equiv \frac{\sum_{i} m_i C_{\text{p},i}}{hA} \tag{7}$$

Fitting the data in **Figure 2**b, τ_{s} was found to be 295.62 s from the heating curve. The heat transfer coefficients (*hA*) was calculated to be 10.79 mW/K according to eq 7 (C_{Cu7S4} = 76.838 J mol^{-1} K^{-1}, m_{Cu7S4} = 5 mg).

$$hA = \frac{\sum_{i} m_i C_{\text{p},i}}{\tau_{\text{s}}} = \frac{C_{\text{Cu}_7\text{S}_4} \cdot m_{\text{Cu}_7\text{S}_4} + C_{\text{CH}_2\text{Cl}_2} \cdot \rho_{\text{CH}_2\text{Cl}_2} \cdot V_{\text{CH}_2\text{Cl}_2} / M_{\text{CH}_2\text{Cl}_2}}{\tau_{\text{s}}}$$

$$= \frac{76.838 \times 0.005 / 159.2 + 102.3 \times 1.325 \times 2.0 / 84.93\text{J/K}}{295.62\text{s}} = 10.79 \text{ mW/K}$$

Thus, the photothermal transduction efficiency of the Cu_7S_4@ZIF-8 nanostructures was calculated to be 31.1% under the irradiation of 1450 nm laser (T_{max} - T_{surr} = 19.7 K from **Figure 2**b):

$$\eta = \frac{hA\left(T_{\max} - T_{\text{surr}}\right) - Q_{\text{in,surr}}}{I\left(1 - 10^{-A_{\lambda}}\right)} = \frac{10.79 \times 19.7 - 57.3}{500 \times \left(1 - 10^{-6.3}\right)} = 31.1\%$$

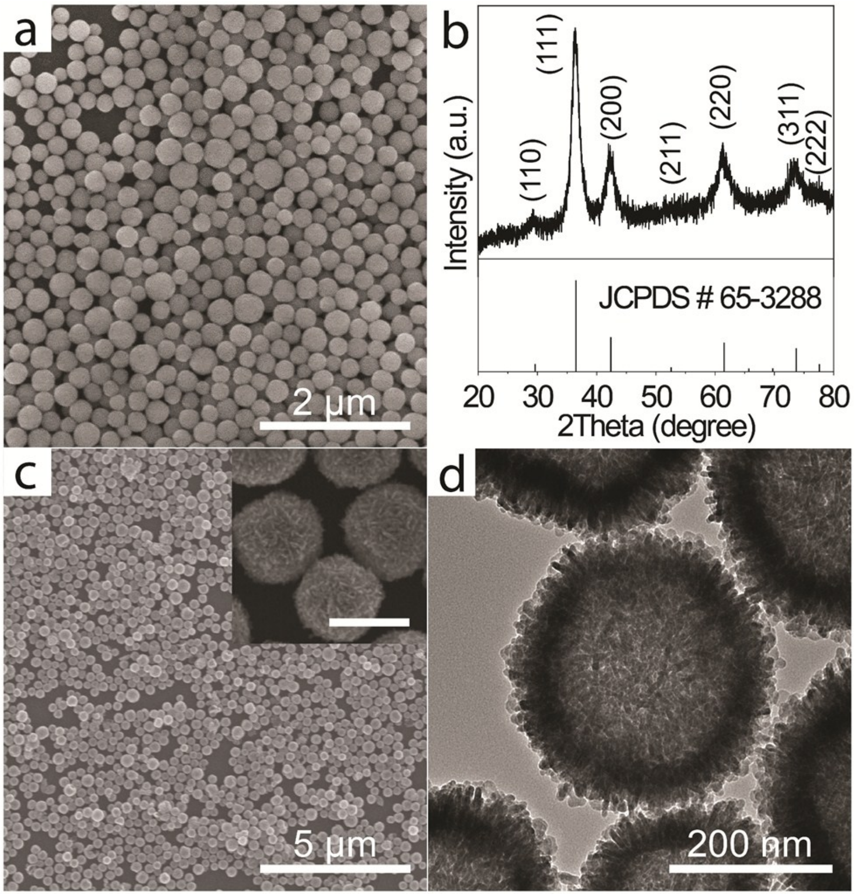

Figure S1. (a, b) SEM image and powder XRD spectra of Cu_2O nanospheres. **(c, d)** SEM and TEM images of hierarchical Cu_7S_4 hollow microspheres. Scale bar in the inset of **(c)** represents 300 nm.

The core-shell Cu_7S_4@ZIF-8 nanostructures were prepared by encapsulating the pre-synthesized Cu_7S_4 NPs with the microporous MOFs. The hierarchical Cu_7S_4 NPs were formed by sulfidation of the polycrystalline Cu_2O precursors. The diameter of the Cu_7S_4 shell (320 ± 50 nm) was slightly larger than that of the Cu_2O nanoparticle (283 ± 46 nm), which can be explained by the faster diffusion speed of O^{2-} ions than that of S^{2-} ions.[1]

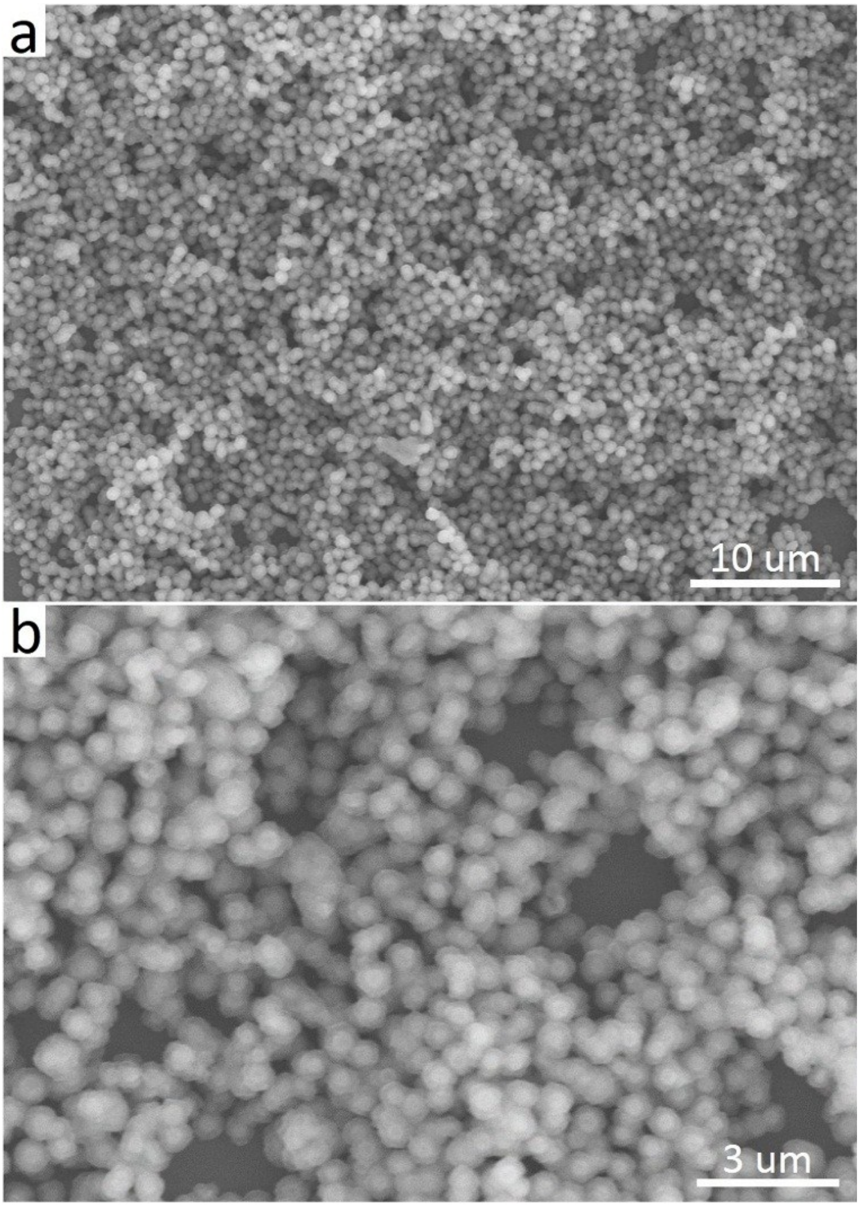

Figure S2. Large area SEM image **(a)** of the core-shell Cu_7S_4@ZIF-8 nanostructures with the magnified view **(b)**. The uniform encapsulation of MOF shells on single Cu_7S_4 NPs indicated the well-controlled crystallization process was achieved by tuning the amount of MOF precursor and the core concentration.

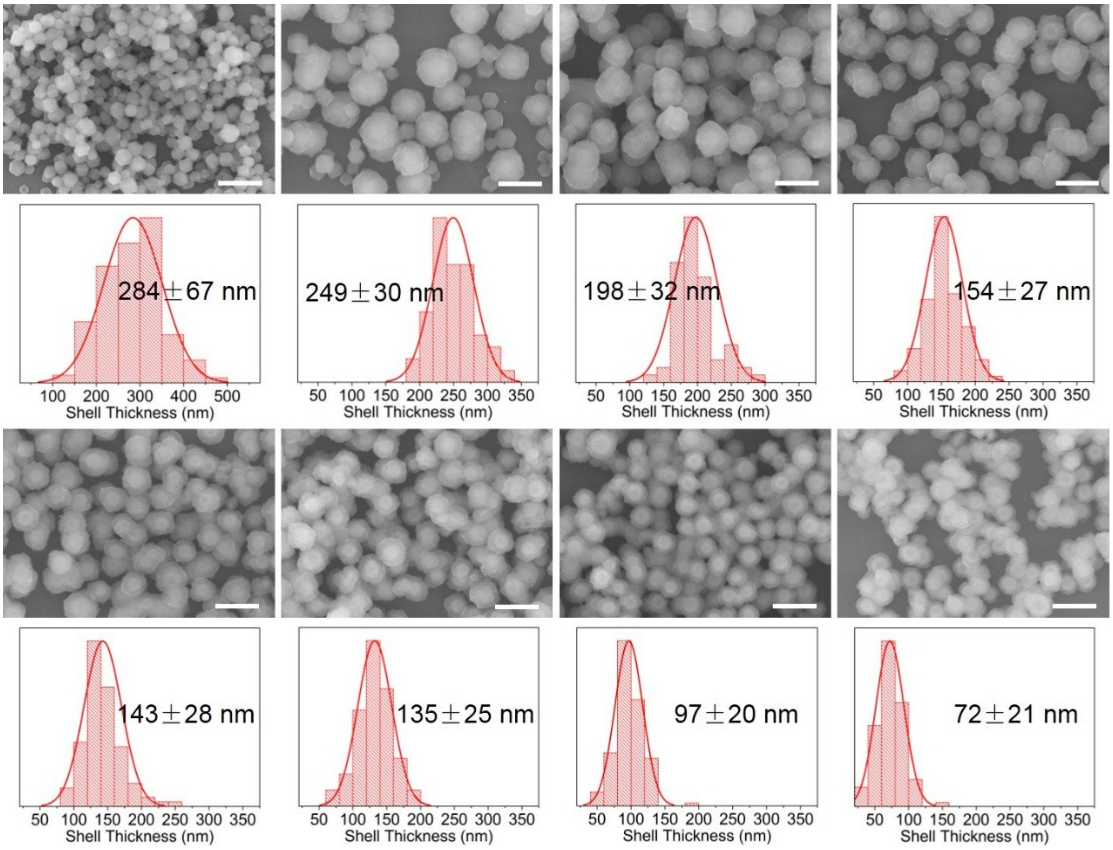

Figure S3. Thickness evolution of the ZIF-8 shells with different Cu_7S_4 core concentration of 0, 12.5, 25, 37.5, 50, 62.5, 75, and 100 mg/L. Scale bar = 1 μm. The shell thickness showed a negative relationship with the core concentration. The homogeneous nucleation of ZIF-8 was obvious at low concentration of the Cu_7S_4 core. While an excessive core concentration caused the aggregation of the core-shell NPs.

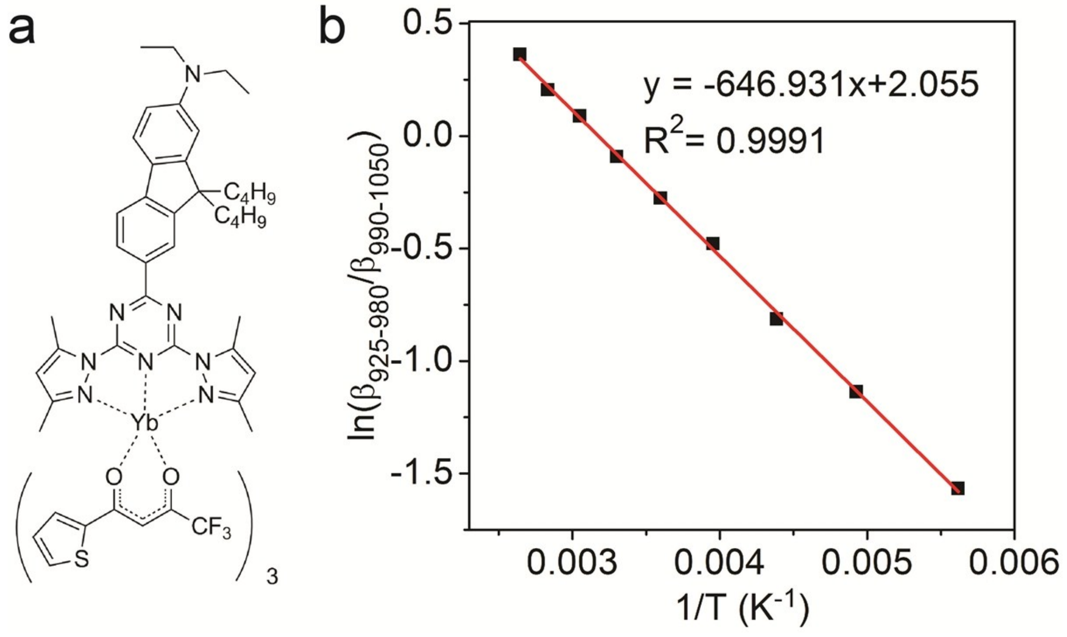

Figure S4. (a) Molecular formula of the Yb^{3+} complex. **(b)** Standard curve of the temperature sensing of the Yb^{3+} complex. The Yb^{3+} luminescence spectrum branching coefficient (β) for different wavelength ranges is defined as the area under the corresponding contour divided by the entire area of the Yb^{3+} emission spectrum. The natural logarithm $\ln(\beta_{925\text{-}980}/\beta_{990\text{-}1050})$ linearly decreases with the increase in the reciprocal of temperature (1/T).

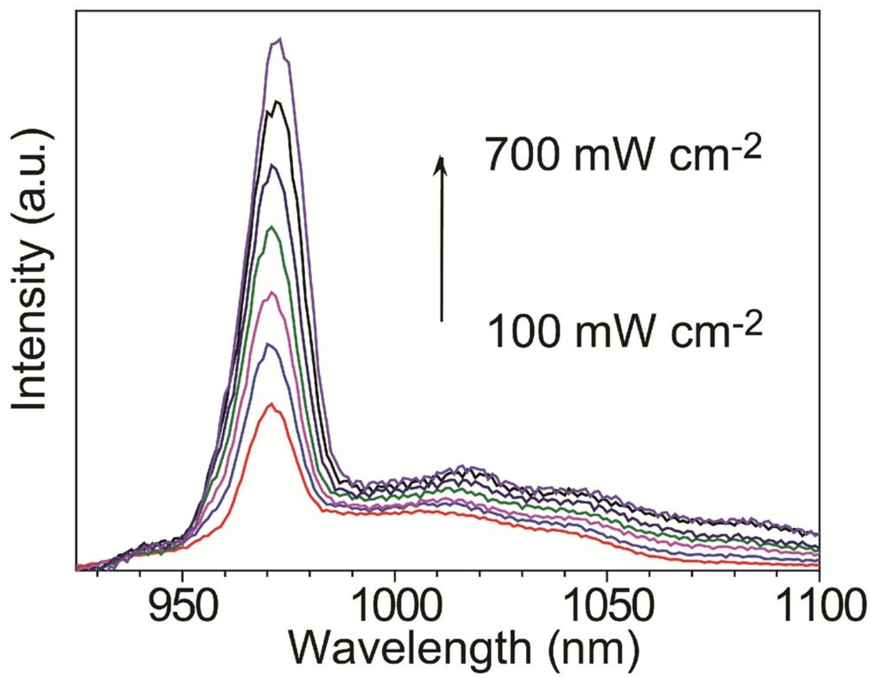

Figure S5. Luminescence spectra of Yb^{3+} complex absorbed on the surface of the Cu_7S_4 NPs under irradiation of 1450 nm laser with different power densities (100, 200, 300, 400, 500, 600, 700 mW cm^{-2}).

Table S1. Temperature Measurement Using Yb^{3+} Complex as Sensor at the Irradiation of 1450 nm Laser.[a]

Laser power (mW)	ΔT_{probe} (°C)	ΔT_{sample}[b] (°C)	T_{sample}[b] (°C)
100	0.4	37.0	61.0
200	1.0	51.0	75.0
300	1.6	64.0	88.0
400	2.1	78.0	102.0
500	2.5	93.0	117.0
600	2.9	109.0	133.0
700	3.4	122.0	146.0

[a] The 1450 nm laser with different intensities was irradiated for 1 min before the collection of the luminescence spectra. [b] The temperature larger than 120°C was calculated by the extrapolation of the standard curve (see **Figure S4**b).

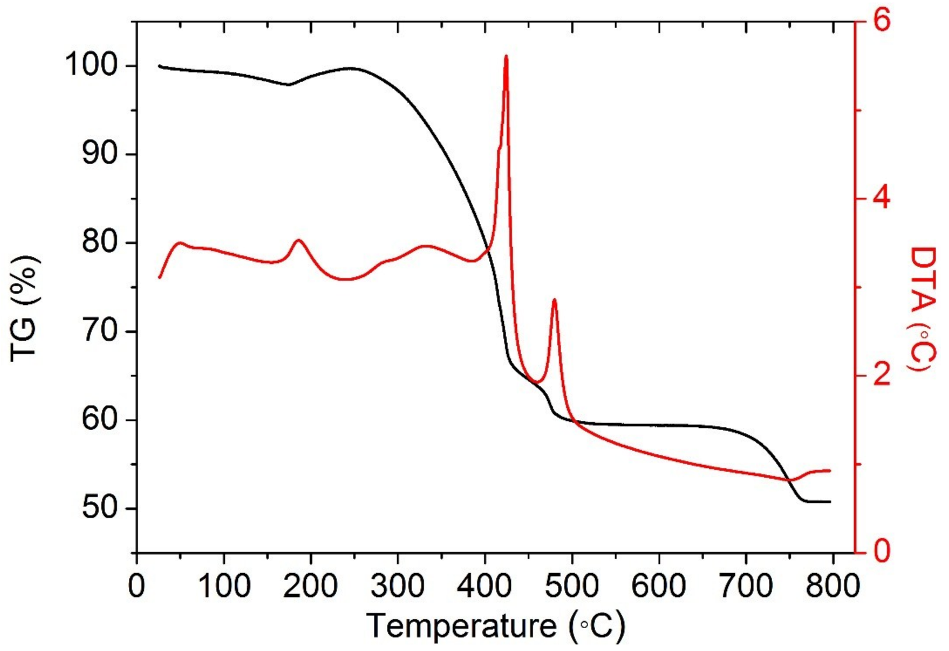

Figure S6. TG and DTA curves of core-shell Cu_7S_4@ZIF-8 samples. The weight-loss around 190°C was attributed to the partial oxidation of the hierarchical Cu_7S_4 microsphere. The ZIF-8 shell showed a good structure stability up to 250°C in the air atmosphere, which is consistent with the previous work.[3]

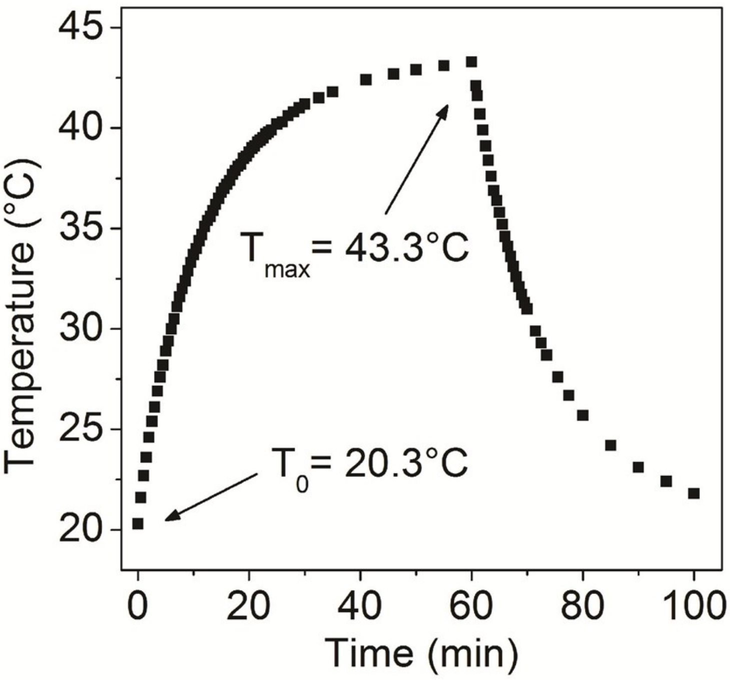

Figure S7. Temperature profile of the core-shell Cu_7S_4@ZIF-8 NPs dispersed in reaction solutions during the laser irradiation (1450 nm, 500 mW). The highest temperature (bulk) of 43.3^{o}C was reached in the catalysis system.

As the Yb^{3+} experiment shown, the surface temperature (~120°C) of the Cu_7S_4 nano-heater is much higher than the temperature (43°C) of the bulk reaction solution. The closer to the nano-heater surface, the higher temperature the reactants can sense. Additionally, the large T gradient around the photothermal core may facilitate the products diffusion in the crevices of the MOF shell. The local enhancement is defined to evaluate the localized heating effect on the catalytic performance in the vicinity of the Cu_7S_4 surface. The total enhancement includes the localized and bulk heating effects. The calculation of local and total enhancement factor is:

$$E_{local} = \frac{Conv_{Photothermal}^{20°C}}{Conv_{Thermal}^{43°C}}$$

$$E_{total} = \frac{Conv_{Photothermal}^{20°C}}{Conv_{Thermal}^{20°C}} = \frac{Conv_{Photothermal}^{20°C}}{Conv_{Thermal}^{43°C}} \cdot \frac{Conv_{Thermal}^{43°C}}{Conv_{Thermal}^{20°C}} = E_{local} \cdot E_{bulk}$$

Table S2. Summary of [3+3] Cyclocondensation Reaction Performance for Cu_7S_4@ZIF-8 NPs at Different Conditions.

Heating method	Light condition	Temperature (°C)	Reaction time (h)	Conversion efficiency (%)
Photothermal	1450 nm laser, 500 mW cm^{-2}	RT	2	47.4 ± 4.8
		RT	4	72.3 ± 6.5
		RT	6	97.2 ± 3.5
Thermal	Dark	20	2	10.6 ± 3.2
		20	4	14.5 ± 3.7
		20	6	18.1 ± 4.4
		43	2	30.0 ± 3.1
		43	4	43.6 ± 5.2
		43	6	55.4 ± 5.5
		43	12	73.2 ± 7.0
		43	18	93.5 ± 4.5

Calculation of the Reaction Enthalpy and Apparent Activation Energy for [3+3] Cyclocondensation Reaction

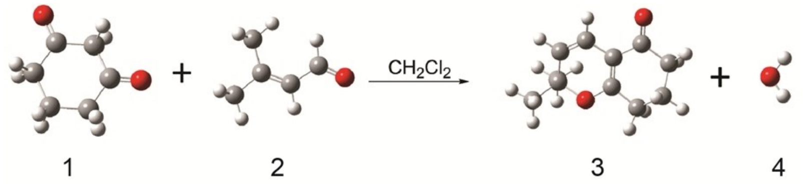

We implemented our geometry optimizations with Gaussian 09 software. During calculations, the Cartesian coordinate is chosen in the input files with symmetry reserved for each molecule. The b3lyp/6-31g basis set is adopted because it is adequate to ensure an accurate results in our calculations. Each molecule is relaxed to its lowest energy state in vacuum. Afterwards, the reaction enthalpy ΔH is estimated by comparing the energy difference between products and reactants, where $\Delta H = \Delta H_p - \Delta H_r$. Though no solvent effects are considered during our calculations, the geometry optimization results are still reasonable to mimic the real system for ΔH in fact is the total bond energy change, which is solvent irrelevant. The results were listed in the following table and the reaction enthalpy ΔH was calculated to be -370 kJ mol^{-1}.

Table S3. Parameters of the Enthalpy Calculation for [3+3] Cyclocondensation Reaction.

Entry	1	2	3	4	ΔH
Energy/a.u.[a]	-383.7853	-270.4776	-578.0178	-76.3861	-0.1410

[a] 1 a.u. = 1 hartree = 4.359810e^{-18} J.

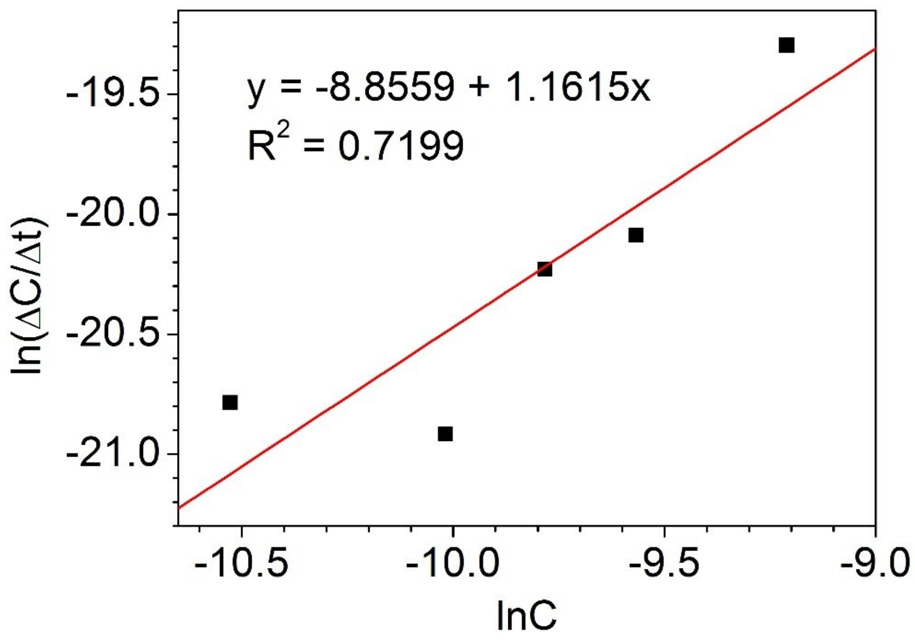

Figure S8. Determination of reaction order of the catalyzed [3+3] cyclocondensation reaction.

For the determination of reaction order of the [3+3] cyclocondensation reaction, the kinetics data of the thermal processes were fitted according to the function:

$$\text{Rate} = \frac{\Delta C}{\Delta t} = kC_{0,i}^{n} \qquad (i = 0, 1, 2 \cdots)$$

$$\ln\frac{\Delta C}{\Delta t} = \ln k + n \ln C_{0,i} \qquad (i = 0, 1, 2 \cdots)$$

The fitting results (**Figure S8**) revealed that the [3+3] cyclocondensation reaction was a pseudo first order reaction. The rate constant k of the thermal process at 43 ^{o}C and 20 ^{o}C was extracted via this fitting method to be e^(-8.8559) and e^(-12.0494), respectively. The apparent activation energy E_a was estimated to be 107 kJ mol^{-1}.

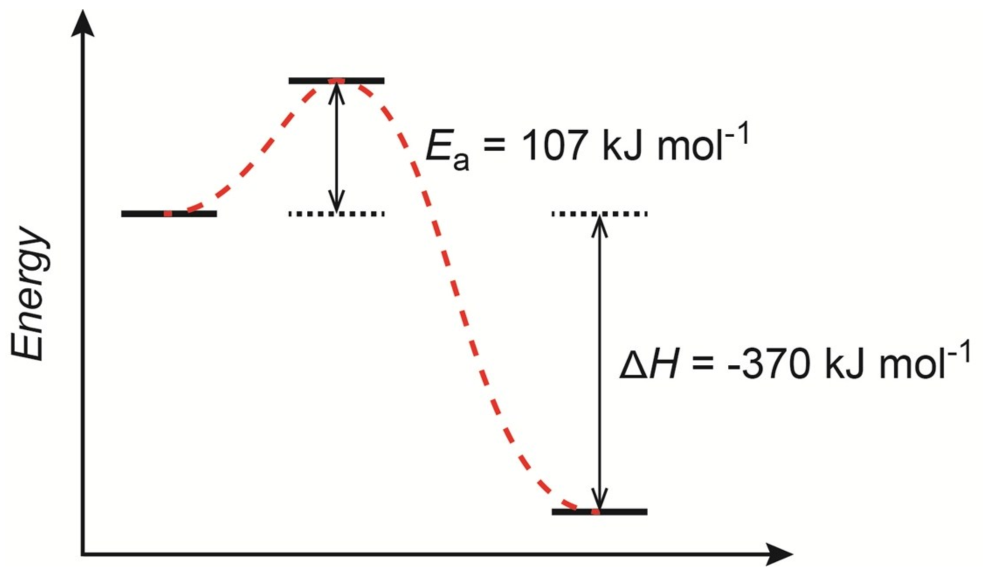

Figure S9. Schematic energy diagram of the catalyzed [3+3] cyclocondensation reaction.

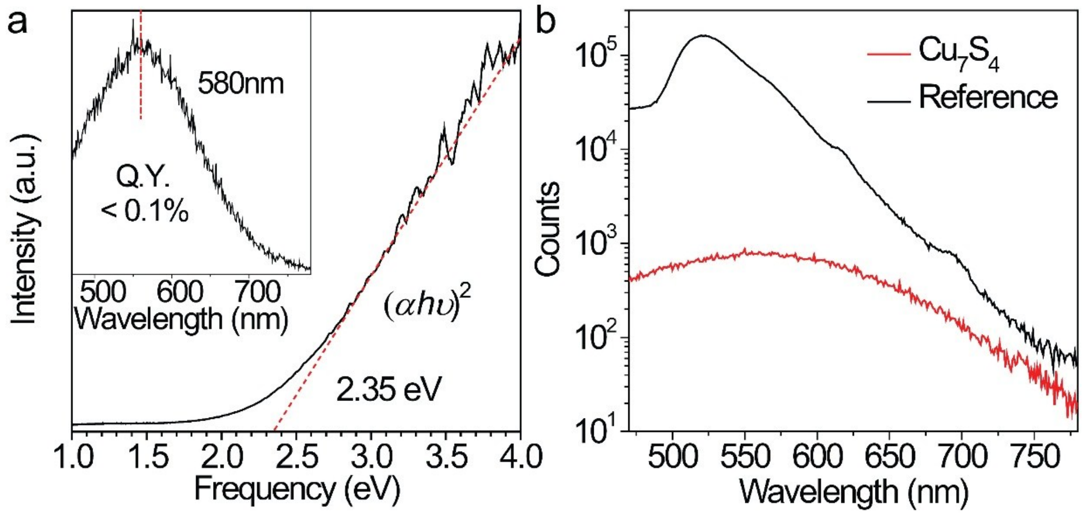

Figure S10. (a) Ultraviolet-visible (UV-Vis) absorption and photoluminescence (PL) spectra (inset) of the hierarchical Cu_7S_4 nanostructures. **(b)** Fluorescence quantum yield measurement of the Cu_7S_4 NPs. The black curve showed the PL spectra of the reference sample with known quantum yield. All PL spectra were recorded at the excitation wavelength of 450 nm.

The PL peak wavelength (580 nm) showed a red-shift against the onset absorption (2.35 eV).[6,7] The PL quantum yield of the hierarchical Cu_7S_4 NPs can calculated according to a reference sample (2,4-dichloro-6-[p-(N,N-diethylamino) biphenylyl]-1,3,5-triazine, DBQ) with known quantum yield.[8]

$$\varphi_S = \frac{A_R}{A_S} \cdot \frac{F_S}{F_R} \cdot \varphi_R = \frac{0.042}{0.326} \times \frac{128134}{9.98 \times 10^6} \times 17.2\% = 2.8 \times 10^{-2}\%$$

Such a low quantum yield indicates the poor luminescence of the Cu_7S_4 NPs. From the perspective of the structure, the extensive grain boundaries between the nanocrystals facilitated the conversion of photo-generated electrons and holes into heat, indicating that the hierarchical Cu_7S_4 NP was a promising photothermal semiconductor for the utilization of full solar spectrum.

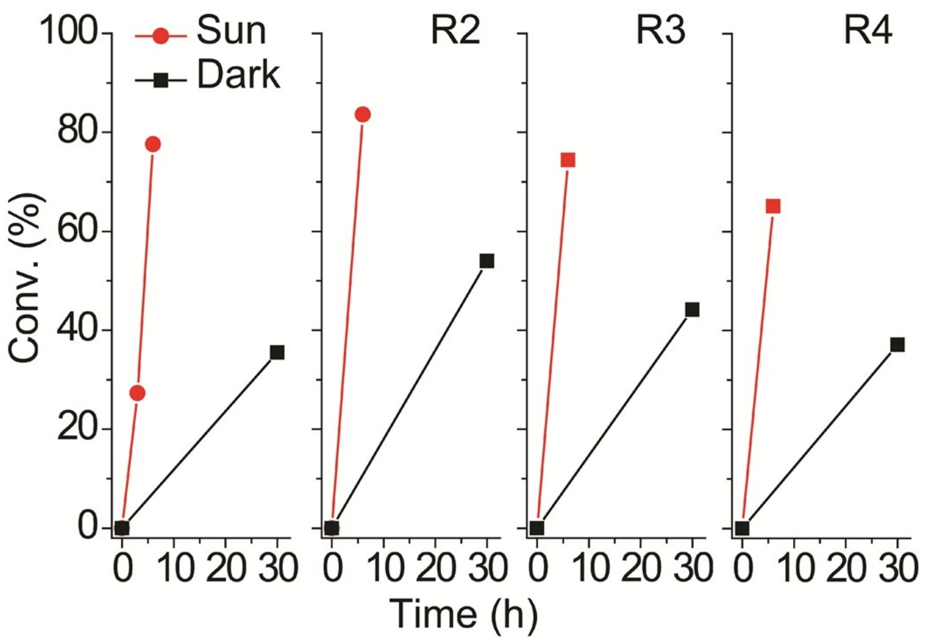

Figure S11. Catalytic performance of the core-shell Cu_7S_4@ZIF-8 NPs with or without the simulated sunlight illumination. Reaction conditions: 100 mW cm^{-2} full-spectrum irradiation, 4.9 cm^2 illumination area, 10 mL reactor, room temperature.

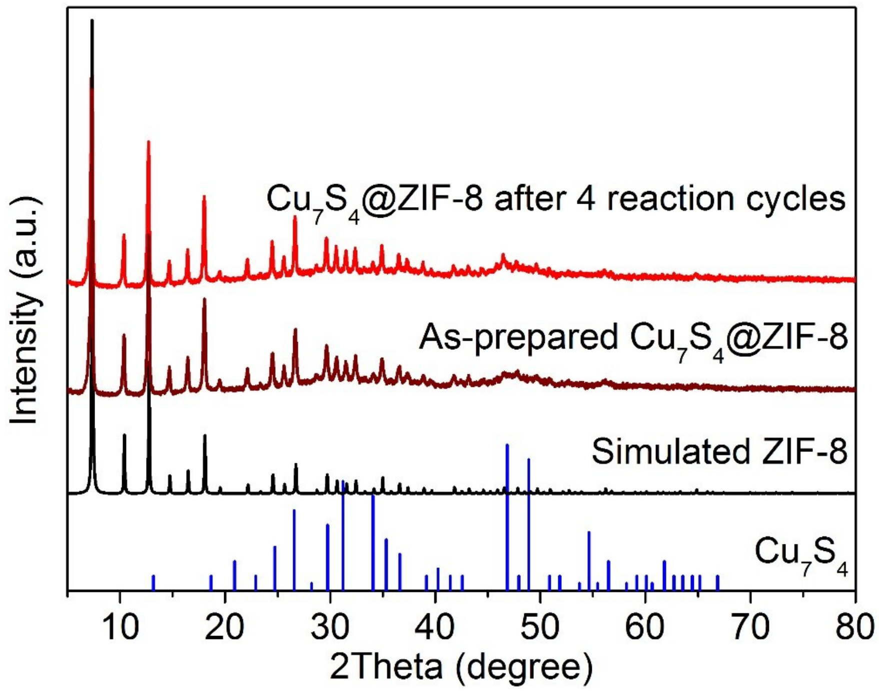

Figure S12. Powder XRD patterns of simulated Cu_7S_4 (JCPDS No. 23-958), simulated ZIF-8, as-prepared Cu_7S_4@ZIF-8 and experimental Cu_7S_4@ZIF-8 after 4 photothermal catalysis cycles for the cyclocondensation reaction.

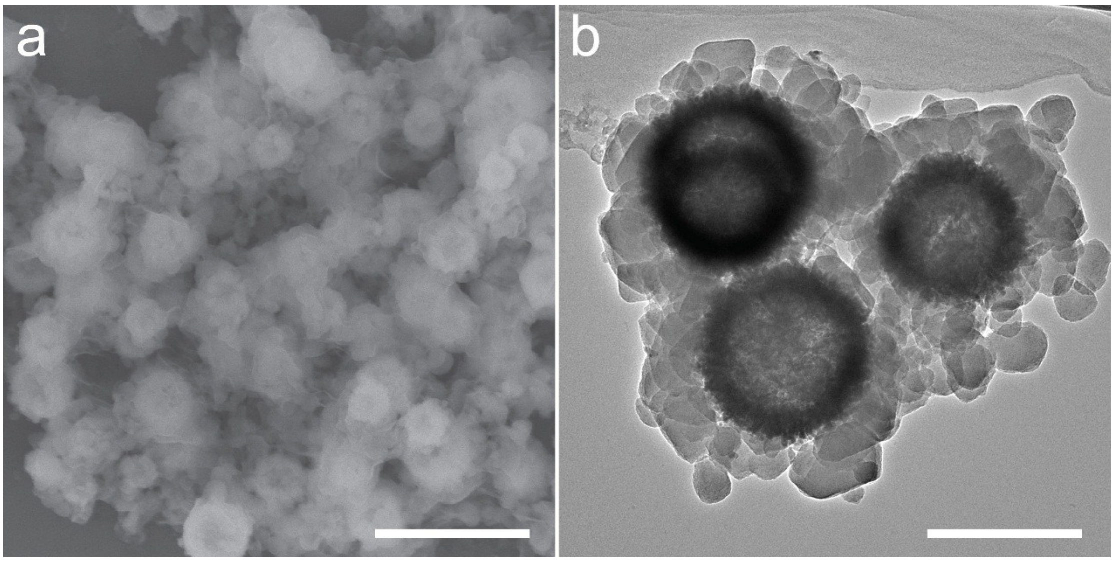

Figure S13. SEM (a) and TEM (b) image of Cu_7S_4@ZIF-8 catalysts before and after 4 consecutive cycles of photothermal catalytic reactions. Scale bar represents 1 μm for (a) and 300 nm for (b), respectively.

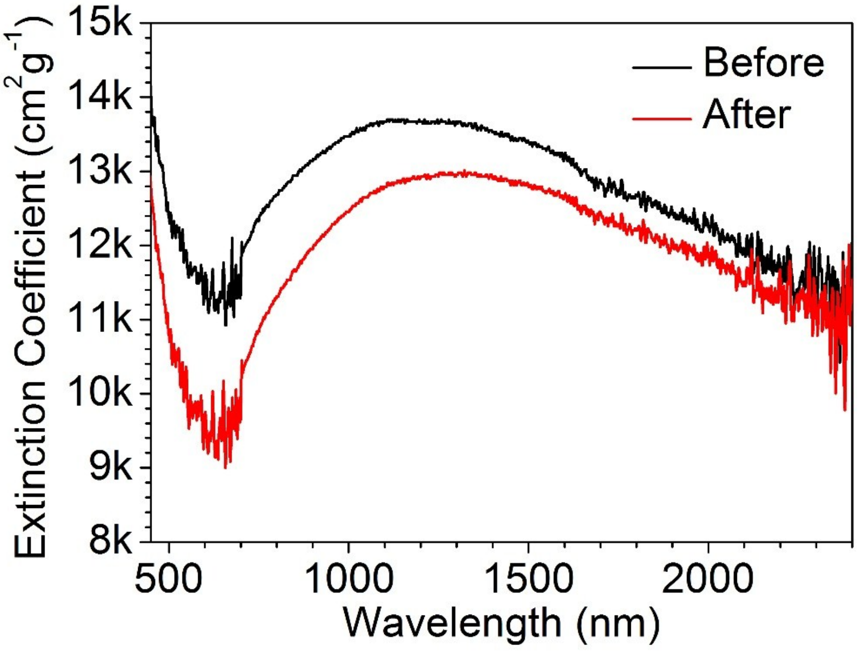

Figure S14. Extinction spectra of Cu_7S_4@ZIF-8 catalysts before and after 4 consecutive cycles of cyclocondensation reactions under simulated sunlight irradiation.

References

1. S. H. Jiao, L. F. Xu, K. Jiang and D. S. Xu, *Adv. Mater.* **2006**, *18*, 1174.

2. S. L. Xiong and H. C. Zeng, Angew. Chem. Int. Ed. **2012**, 51, 949.

3. F. Zhang, Y. Y. Wei, X. T. Wu, H. Y. Jiang, W. Wang and H. X. Li, *J. Am. Chem. Soc.* **2014**, *136*, 13963.

4. D. K. Roper, W. Ahn and M. Hoepfner. *J. Phys. Chem. C* **2007**, *111*, 3636.

5. C. M. Hessel, V. P. Pattani, M. Rasch, M. G. Panthani, B. Koo, J. W. Tunnell and B. A. Korgel. *Nano Lett.* **2011**, *11*, 2560.

6. M. Behboudnia and B. Khanbabaee. *J. Cryst. Growth* **2007**, *304*, 158.

7. S.-C. Liufu, L.-D. Chen, Q. Yao and F.-Q. Huang. *J. Phys. Chem. C* **2008**, *112*, 12085.

8. Y. Ma, R. Hao, G. S. Shao and Y. Wang, *J. Phys. Chem. A* **2009**, *113*, 5066.
